# Supplementary material for: Scalable Memdiodes Exhibiting Rectification and Hysteresis for Neuromorphic Computing
Source: Sci Rep. 2018 Aug 28;8:12935. doi: 10.1038/s41598-018-30727-9 (PMC6113211; doi:10.1038/s41598-018-30727-9)
Supplement: Supplementary file 1 — Supplementary Information [file 41598_2018_30727_MOESM1_ESM.docx]

Scalable Memdiodes Exhibiting Rectification and Hysteresis for Neuromorphic Computing

Supplemental Information

Joshua C. Shank^1^, M. Brooks Tellekamp^1^, Matthew J. Wahila^2^, Sebastian Howard^2^, Alex S. Weidenbach^1^, Bill Zivasatienraj^1^, Louis F. J. Piper^2^, and W. Alan Doolittle^1^*

^1^ Department of Electrical and Computer Engineering, Georgia Institute of Technology, Atlanta, GA 30332, United States of America

^2^ Department of Physics, Applied Physics and Astronomy, Binghamton University, Binghamton, NY 13902, United States of America

*alan.doolittle@ece.gatech.edu

**XPS Analysis Details**

**
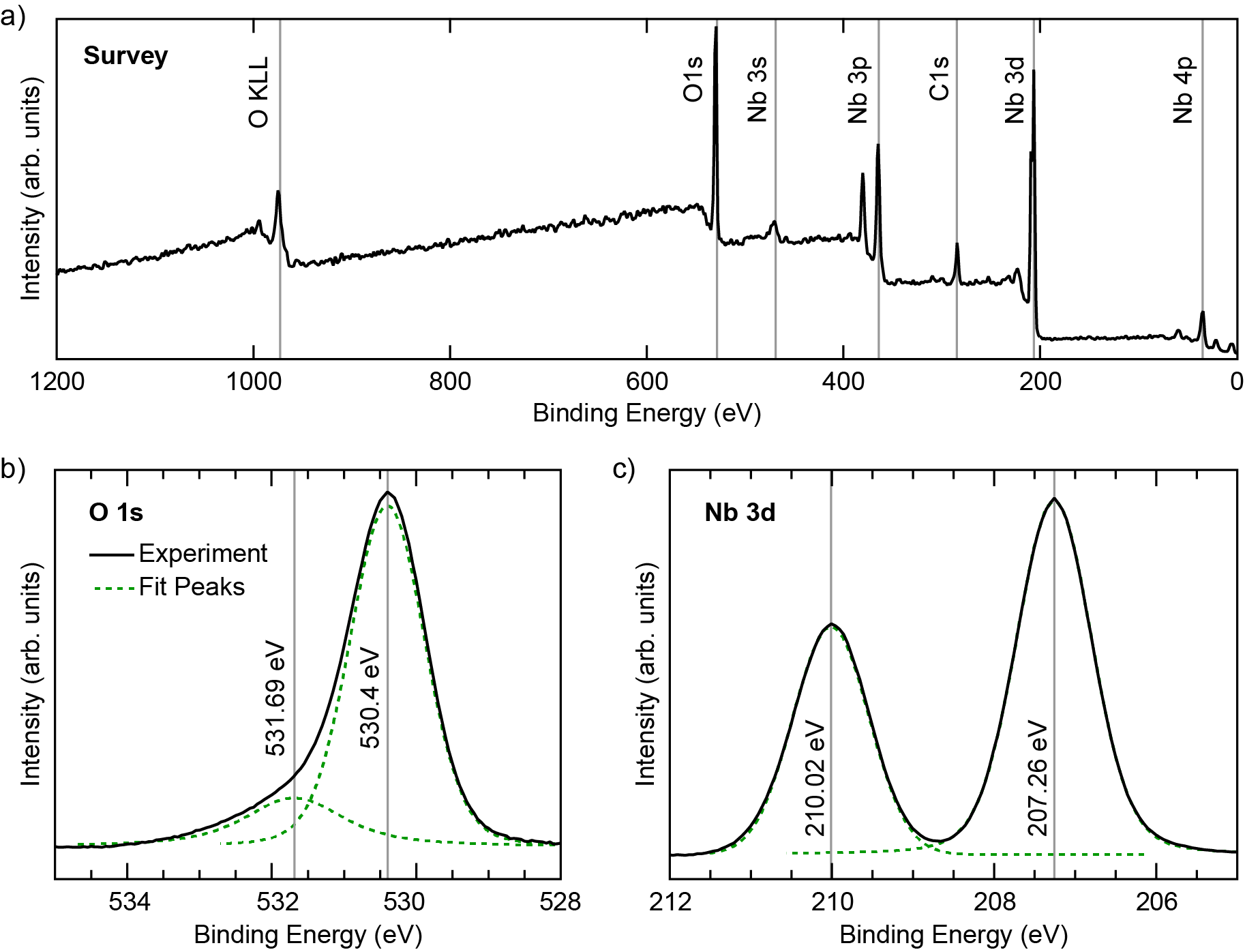
 Figure S1. Representative XPS spectra.** a) Representative XPS survey scan of a Nb_2_O_5_ film grown at 40 W. b) Averaged O 1s and c) averaged Nb 3d core regions of Nb_2_O_5_ films grown at 40 W showing representative XPS peak fitting analysis.

A total of 10 samples of Nb_2_O_5_ grown at 30, 40 and 50 W Nb target power were measured using XPS at Binghamton University. A total of 18 different spots were measured for each of the 50 W and 30 W samples, while 17 spots were measured for the 40 W films. The O 1s, Nb 3d and the C 1s were measured for each spot. A representative survey scan is shown in Fig. S1a.

To overcome the insulating character of the films, charge compensation was employed consisting of an electron gun and low-energy Ar ions. The C 1s peak at 284.8 eV (i.e., adventitious carbon) was then used as an internal reference for energy calibration. Peak fitting analysis was performed using a Shirley background and pseudo-Voigt line profiles for the C 1s, O 1s, and Nb 3d peaks, representative peak fits are shown in Fig. S1b and c.

**Figure S2.** Preferential sputtering in XPS depth profiles of sputtered Nb_2_O_5-x_.

As shown in Figure S2, the differing sputter yields of Nb and O make compositional depth-profile analysis of the sputtered Nb_2_O_5-x_ difficult to interpret. To remove ambiguity films were vacuum packed immediately following deposition and stored under dry nitrogen until characterization. Sample mounting for characterization was performed in an Ar glovebox to prevent surface contamination. Despite these precautions a surface oxide was observed. This surface oxide was fit and removed, as is shown in the main text and Table S3.

**Table S1. Core level peak positions from XPS.** Average O 1s, C 1s, and Nb 3d peak positions for 30, 40, and 50 W depositions determined from XPS measurements, as well as the average positions including all deposition powers.

|  | | Average Peak Location (eV) | | | |
| --- | --- | --- | --- | --- | --- |
|  |  | 30W | 40W | 50W | Average |
| O 1s | Peak 0 | 530.41 ± 0.13 | 530.37 ± 0.23 | 530.42 ± 0.11 | 530.40 ± 0.16 |
|  | Peak 1 | 531.73 ± 0.11 | 531.61 ± 0.25 | 531.72 ± 0.11 | 531.69 ± 0.17 |
| C 1s | Peak 0 | 284.84 ± 0.19 | 284.93 ± 0.49 | 284.80 ± 0.09 | 284.80 ± 0.09 |
|  | Peak 1 | 286.54 ± 0.91 | 286.50 ± 1.12 | 286.09 ± 0.28 | 286.38 ± 0.87 |
|  | Peak 2 | 288.85 ± 0.19 | 288.78 ± 0.25 | 288.86 ± 0.18 | 288.83 ± 0.21 |
| Nb 3d | Peak 0 | 207.32 ± 0.21 | 207.26 ± 0.11 | 207.20 ± 0.10 | 207.26 ± 0.16 |
|  | Peak 1 | 210.08 ± 0.22 | 209.99 ± 0.14 | 209.97 ± 0.09 | 210.02 ± 0.17 |

Table S1 displays the average binding energy locations of the core-level peaks used for the XPS peak fitting. Three peaks were required to fit the C 1s core-level. Two peaks were required for the O 1s core-level, reflecting the bulk Nb_2_O_5_ (530.4 eV) and surface contamination layer (531.7 eV). Two peaks were employed for the Nd 3d core-level to account for the spin-orbit splitting for a Nb^5+^ oxidation state.[1,2] No Nb^4+^ component was required to get good fits.


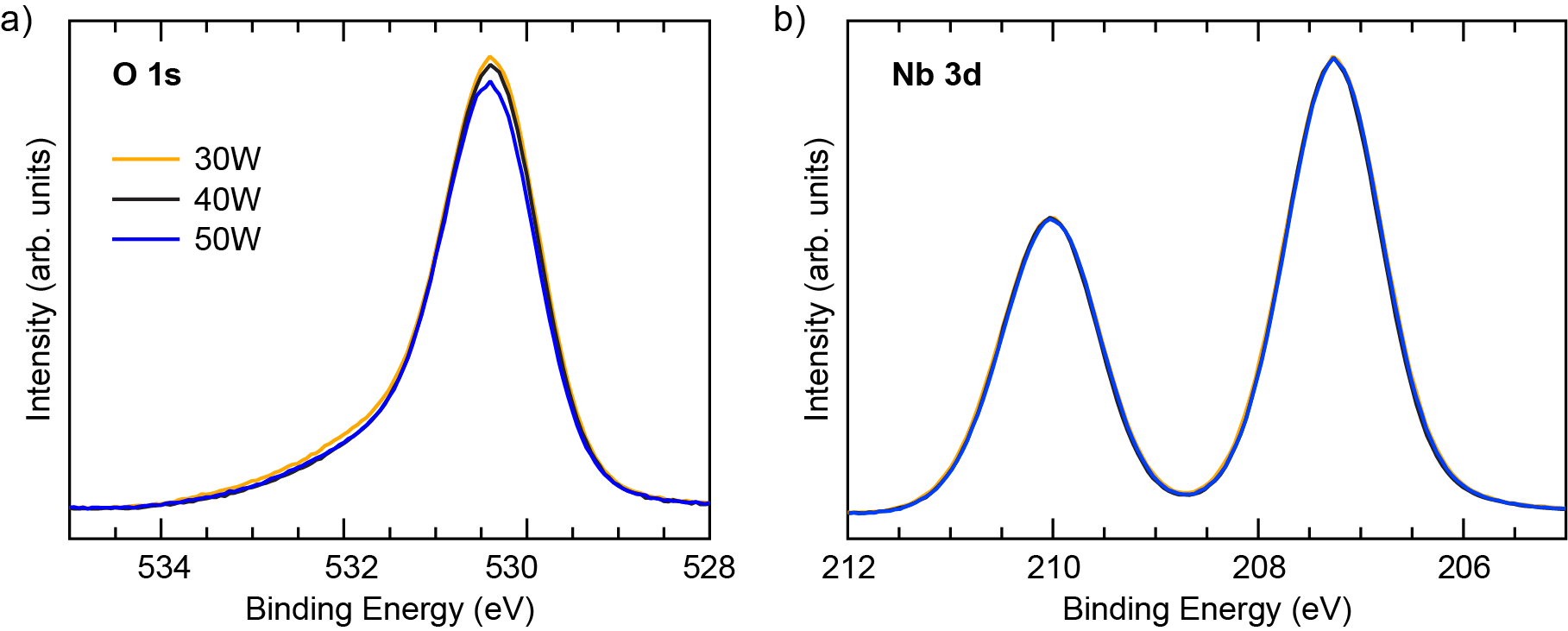


**Figure S3. Average film core level spectra from XPS.** a) Averaged and Shirley background subtracted O 1s and b) Nb 3d core regions of Nb_2_O_5_ films grown using 30, 40, and 50 W deposition powers. Core regions are scaled to normalize the Nb 3d_5/2_ peak.

Figure S3 shows the averaged Nb 3d and O1s core region for the 30, 40, and 50 W depositions. The Nb 3d core regions were scaled to normalize the Nb 3d_5/2_ peak. The same scaling was then applied to the O 1s core region for easy comparison of relative peak height changes. All deposition powers resulted in an identical Nb 3d doublet shape indicating the charge state of the niobium does not change significantly. Meanwhile, the O 1s peak height changes by a small amount between the three deposition powers, indicating a slightly lower oxygen content at higher deposition powers. To properly quantify these compositional changes, the atomic concentration of each element at each deposition power was calculated using the following equation:

$\frac{{A_{cl}}/{{SF}_{cl}}}{\sum_{cl} {A_{cl}}/{{SF}_{cl}}}={at}_{cl}\%$,

where $A_{cl}$ and ${SF}_{cl}$ are the total area and structure factor of a core level.

Table S3 displays the determined atomic concentrations and Nb:O ratios. Despite the care taken to reduce surface contamination, the ~30% carbon measured on all samples is likely the result of the samples’ exposure to air immediately after growth which allowed for carbon and oxygen containing species from the atmosphere to deposit on the film surface. As the probing depth of the XPS is only around 5 nm into the material, care has to be taken to account for this surface contamination during the compositional analysis.

For stoichiometric Nb_2_O_5_, the niobium to oxygen ratio is 0.4. For all deposition powers the Nb:O ratio is below the theoretical 0.4 when including all oxygen in the analysis, indicating that all powers are slightly oxygen rich or niobium deficient. However, if the secondary oxygen peak at higher binding energy is ignored, attributing it to surface contamination species and/or over oxidation at the surface rather than representative of the bulk film, then the analysis results in oxygen deficient compositions as expected. As deposition power increases, the Nb:O ratio increases as expected regardless of the oxygen peaks included in the analysis.

**Table S3. Average film compositions from XPS.** Average total niobium, oxygen, and carbon atomic percentages for 30, 40, and 50 W depositions determined from XPS measurements, as well as Nb:O ratios determined by both including and excluding secondary oxygen peaks attributed to surface species in the compositional analysis.

|  | % Nb | % O | % C | Nb:O Ratio | |
| --- | --- | --- | --- | --- | --- |
|  |  |  |  | With Surface O | Without Surface O |
| 30W | 18.86 ± 2.35 | 50.75 ± 4.73 | 30.40 ± 6.79 | 0.371 ± 0.025 | 0.481 ± 0.027 |
| 40W | 20.22 ± 1.14 | 52.94 ± 1.47 | 26.84 ± 2.38 | 0.382 ± 0.017 | 0.482 ± 0.021 |
| 50W | 19.70 ± 1.06 | 51.15 ± 1.08 | 29.15 ± 1.72 | 0.385 ± 0.020 | 0.497 ± 0.018 |

Shown in Figure S4, the correlations between the different atomic species were investigated to confirm if the secondary oxygen peak is indeed related to surface contamination and not the bulk film. By comparing the atomic percentage of one atomic species to the other species, it can be determined if they are in any way correlated. Atomic species whose percentages are positively correlated are likely to be found in the same film layer, while species that are negatively correlated are likely to be found in different layers. For example, a film with a larger amount of surface contamination will measure a greater percentage of all surface contaminant species, but a smaller percentage of all bulk film species than a film with less contamination.

Shown in Fig. S4a, the Nb and the bulk O are clearly positively correlated. In contrast, as shown in Fig. S4c and e, the Nb has a clear negative correlation to both the C and the surface O. Furthermore, the slopes of the negative correlations are nearly identical, further reinforcing that the Nb is in a separate layer to both the C and surface O. The bulk O also has a negative correlation to the C and surface O with nearly identical slopes, confirming that it is also in a separate layer. Meanwhile the surface O and C are not strongly correlated, indicating that although they are likely in the same surface layer, they are not present in any consistent ratio.


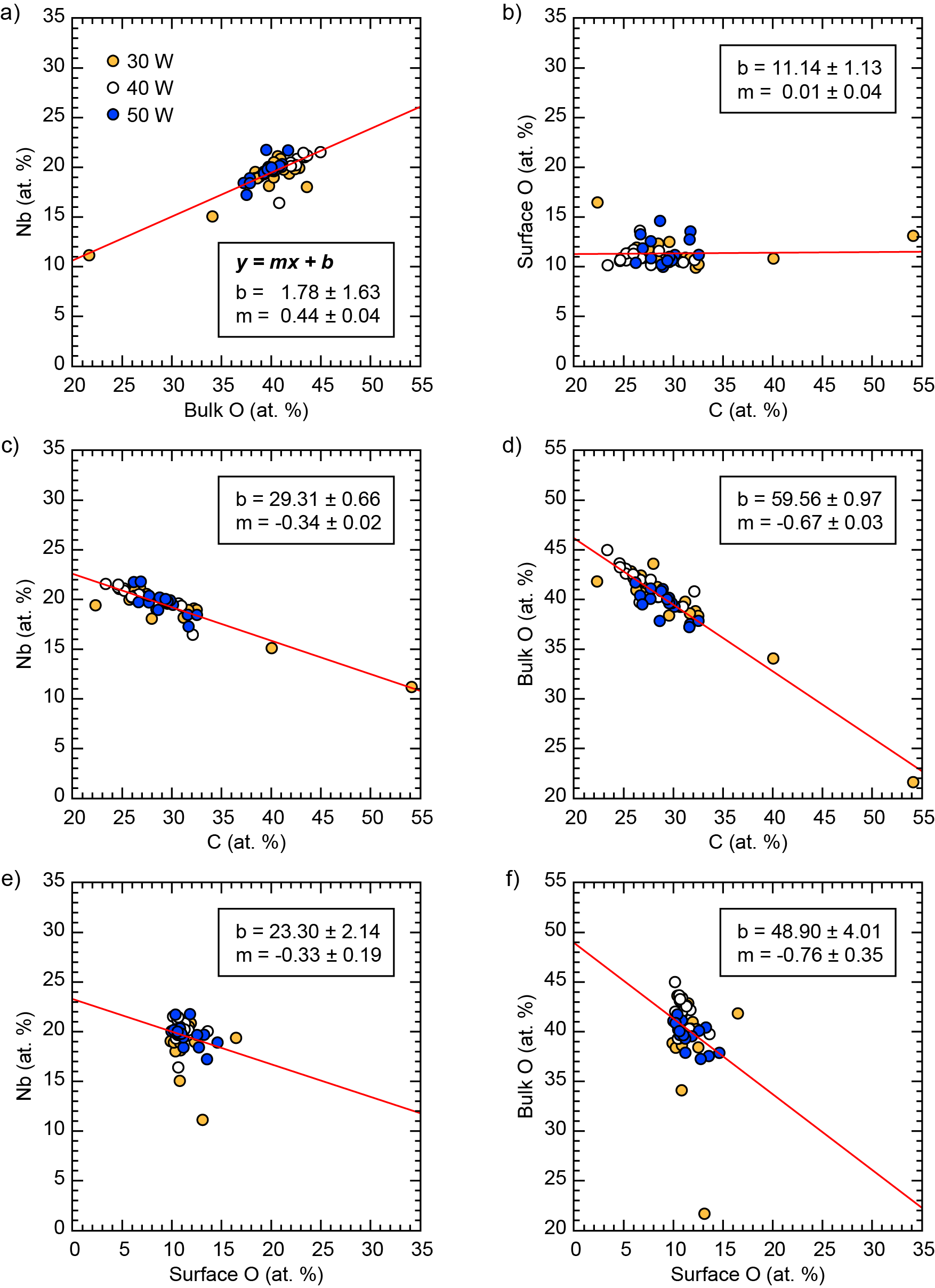


**Figure S4. Correlations between amounts of atomic species.** Total atomic percentage of a) Nb vs. bulk O, b) surface O vs. C, c) Nb vs. C, d) O vs. C, e) Nb vs. surface O, and f) bulk O vs. surface O for 30, 40, and 50 W depositions.

**Other Material Characterization**


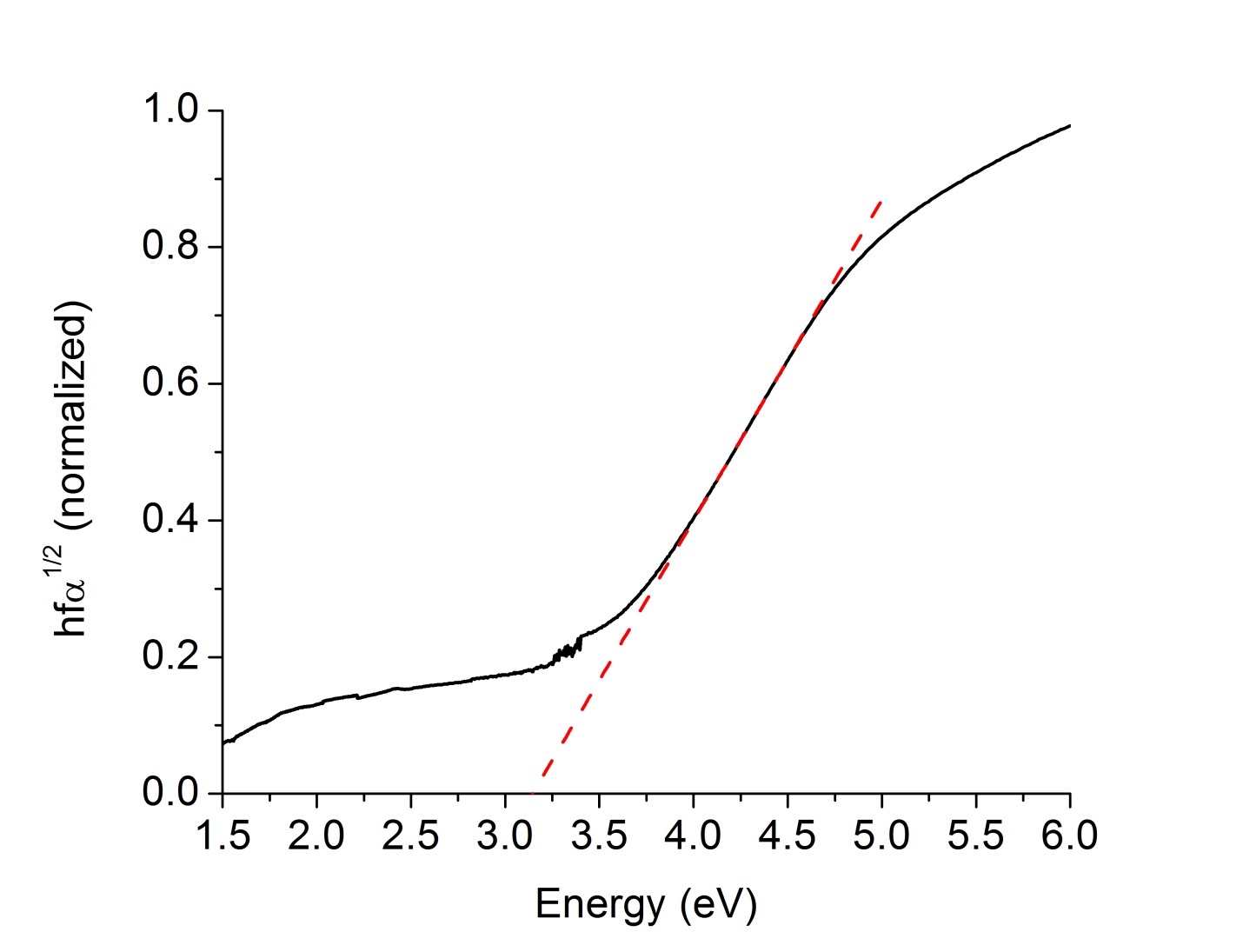


**Figure S5.** The optical band gap as extrapolated by UV-visable spectroscopy is 3.15 +/- 0.004 eV. The measured bandgap is lower than reported range of 3.2 – 4 eV, and was measured by UV-Vis Spectroscopy and plotted using the indirect allowed transition Tauc coefficient (r = 2).[[3](#_ENREF_1)] The lower bandgap correlates with the lower density film, as shown in Figure S7.


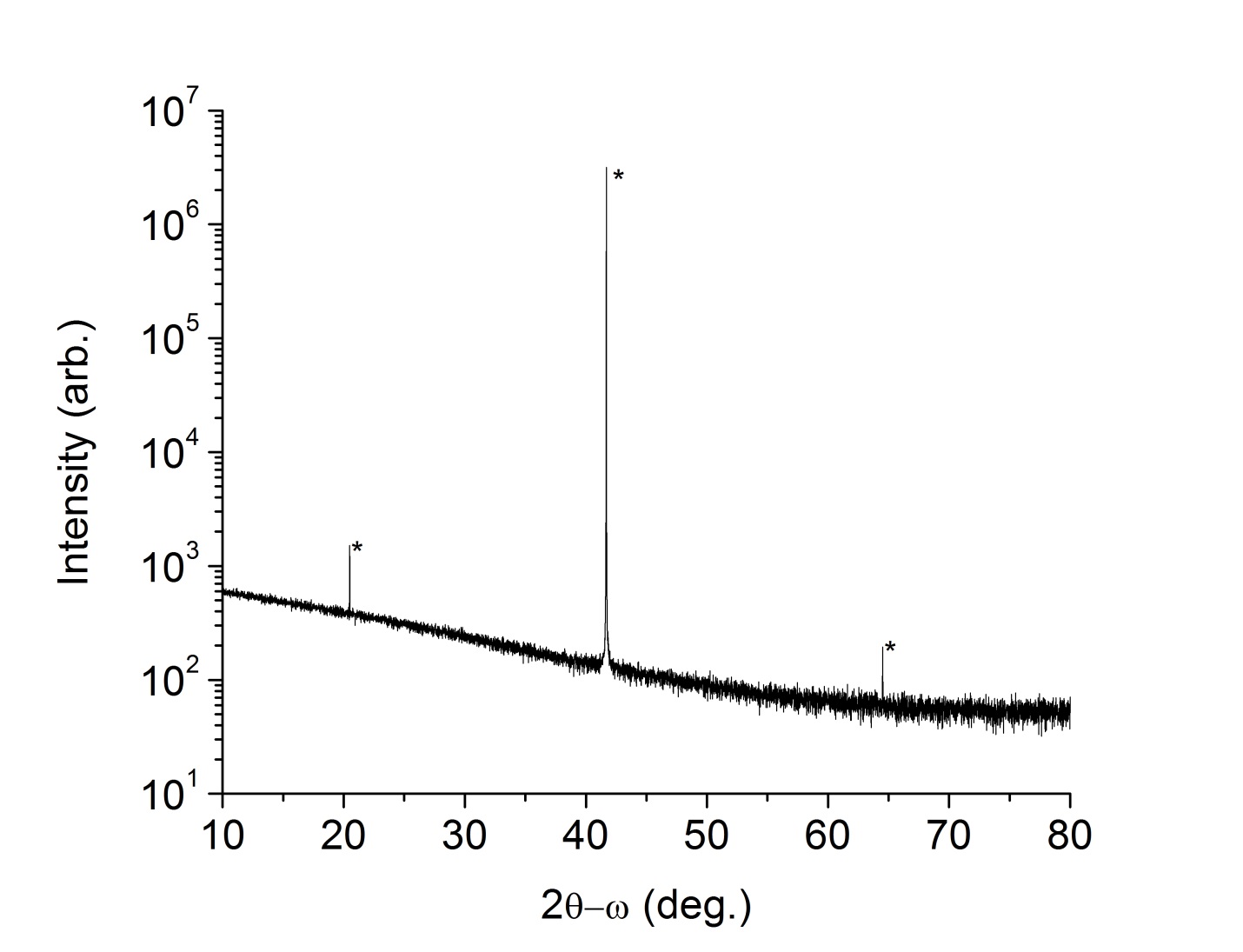


**Figure S6.** XRD shows no peaks other than the sapphire substrate peaks (*) indicating that the deposited niobium oxide is amorphous.


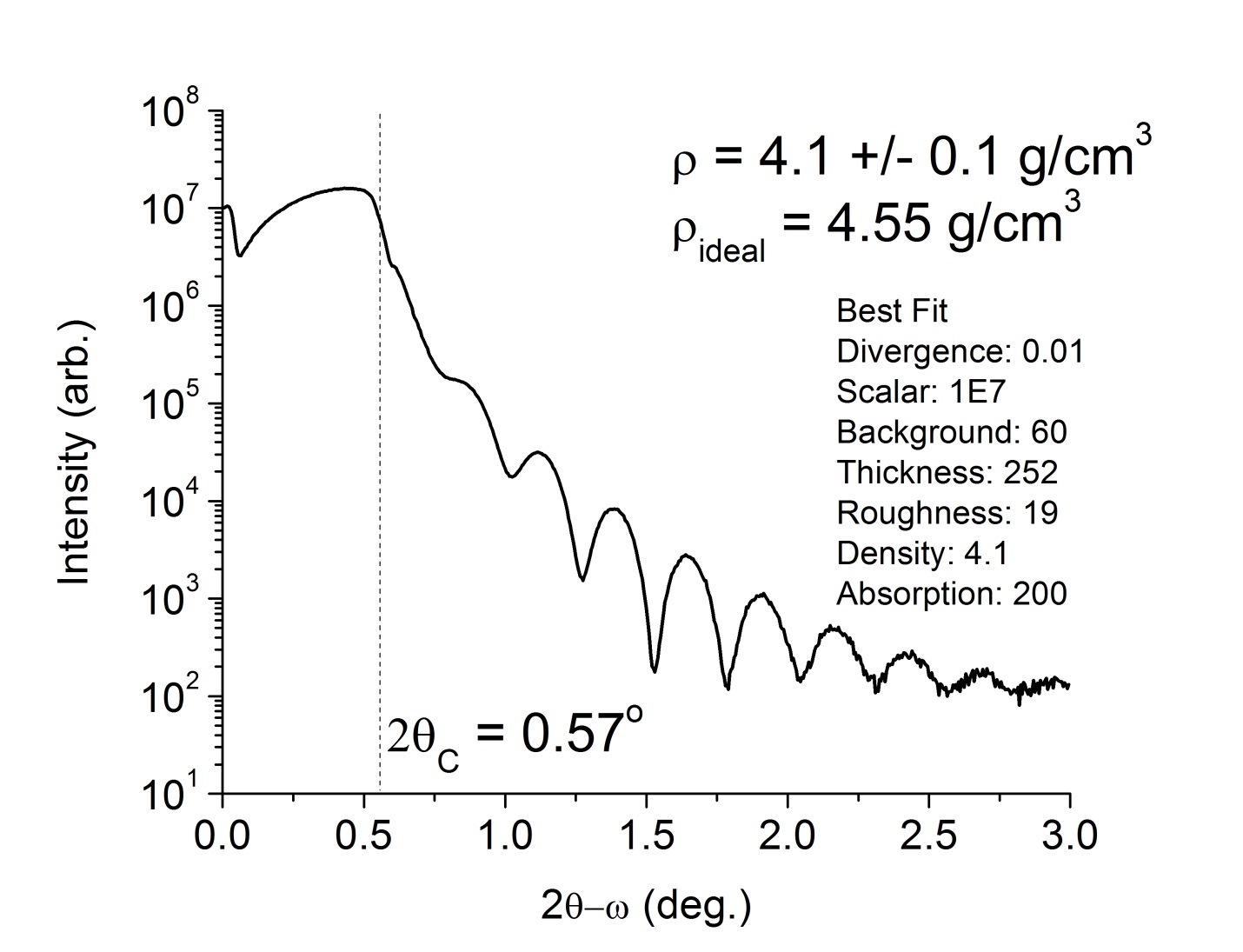


**Figure S7**. Thickness and film density were determined by fitting x-ray reflectivity (XRR) data. Roughness is given in Å RMS, thickness in Å, and density (ρ) in g/cm^3^. ρ_ideal_ is the reported density value for stoichiometric Nb_2_O_5_.

**References**

[1] T. Hryniewicz, K. Rokosz, H.R. Zschommler Sandim, [*SEM/EDX and XPS studies of niobium after electropolishing*](http://www.sciencedirect.com/science/article/pii/S0169433212015905), Applied Surface Science, Volume 263 Pages 357-361 (2012)

[2] Weibin, Z., Weidong, W., Xueming, W., Xinlu, C., Dawei, Y., Changle, S., Liping, P., Yuying, W. and Li, B., *The investigation of NbO_2_ and Nb_2_O_5_ electronic structure by XPS, UPS and first principles methods*. Surf. Interface Anal., 45: 1206–1210. (2013)

[3] J. Dash, L. Chen, M. R. Topka, P. H. Dinolfo, L. Zhang, K. Kisslinger, T.-M. Lu, and G.-C. Wang, "A simple growth method for Nb_2_O_5_ films and their optical properties," *RSC Advances,* vol. 5, pp. 36129-36139, 2015.
